# Exposure to Microplastics during Early Developmental Stage: Review of Current Evidence

**DOI:** 10.3390/toxics10100597

**Published:** 2022-10-10

**Authors:** Nur Hanisah Amran, Siti Sarah Mohamad Zaid, Mohd Helmy Mokhtar, Latifah Abd Manaf, Shatrah Othman

**Affiliations:** 1 Department of Environment, Faculty of Forestry and Environment, Universiti Putra Malaysia (UPM), Serdang 43400, Selangor, Malaysia; 2 Department of Physiology, Faculty of Medicine, Universiti Kebangsaan Malaysia, Kuala Lumpur 56000, Selangor, Malaysia; 3 Department of Molecular Medicine, Faculty of Medicine, Universiti Malaya, Kuala Lumpur 50603, Selangor, Malaysia

**Keywords:** microplastics, early developmental stage, prepubertal development, plastics, human health

## Abstract

In the last few decades, microplastics (MPs) have been among the emerging environmental pollutants that have received serious attention from scientists and the general population due to their wide range of potentially harmful effects on living organisms. MPs may originate from primary sources (micro-sized plastics manufactured on purpose) and secondary sources (breakdown of large plastic items through physical, chemical, and biological processes). Consequently, serious concerns are escalating because MPs can be easily disseminated and contaminate environments, including terrestrial, air, groundwater, marine, and freshwater systems. Furthermore, an exposure to even low doses of MPs during the early developmental stage may induce long-term health effects, even later in life. Accordingly, this study aims to gather the current evidence regarding the effects of MPs exposure on vital body systems, including the digestive, reproductive, central nervous, immune, and circulatory systems, during the early developmental stage. In addition, this study provides essential information about the possible emergence of various diseases later in life (i.e., adulthood).

## 1. Introduction

Plastics have been used extensively in various daily life applications. However, many plastic products are disposed of after a single use, and the improper disposal and elimination of plastics contributes to environmental pollution [1]. In 2010, 4.8–12.7 million tons of plastic litter was deposited into surface waters, and an estimated 100–250 million tons of plastic litter is projected to enter surface waters by 2025 [2]. Asian countries are expected to be the largest producers of plastic products (50%), followed by Europe (19%), North America (18%), the Middle East and Africa (7%), and Latin America (5%) [3]. Moreover, plastic residue in the environment will not decay, but it can be progressively broken down into small particles known as microplastics (MPs) with a size of less than 5 mm.

MPs pollution has been a major global environmental issue. However, no ultimate solution is yet available for successfully managing or reducing the increasing amount of plastics in the environment because of their versatility and convenience for a wide variety of daily usage. Through the years, a significant number of studies have found that a continuous exposure to MPs may induce a wide variety of disruptive effects on the health of many species. In addition, plastic additives, such as bisphenol A (BPA) and phthalates, hold together plastic polymers through weak non-covalent forces, allowing these chemicals to leach easily into the surrounding environment [4].

Given their small size, MPs may bioaccumulate inside living organisms, resulting in various health effects, such as growth and reproduction issues, oxidative stress, inflammation, physical stress, weakened immunity, histological damage, or even death [5,6]. A recent study demonstrated that an exposure to polystyrene MPs on a human intestinal sample may cause DNA damage due to oxidative stress, as confirmed by the comet assay [7]. Moreover, MPs exposure during the developmental stage is detrimental, because epigenetic changes during the fetal stage can cause diseases in adulthood [8].

The effects of MPs exposure during early development are important because rapid growth and development occur during the prepubertal age. Moreover, the developmental stages should be precisely synchronized to ensure complete functionality development. Furthermore, the sex hormone level in the prepubertal phase is relatively lower than that in the pubertal phase, because the neuroendocrine development of the hypothalamus–pituitary–gonadal axis is still immature. Thus, body systems are highly susceptible to the effects of endocrine-disrupting chemicals (EDCs) from various plastic-based products during this sensitive development period [9]. In addition, children have many more years to live than adults; therefore, they will have a longer life span to acquire chronic illnesses caused by early exposure. Many diseases, such as cancer and neurological disorders, are assumed to develop in phases that occur years or even decades from the onset of the conditions. Thus, when assessing the health and social effects of environmental risks on children, including their effects on childhood health and the long-term perspective of health implications over an individual’s life cycle is important [10,11].

A previous study demonstrated that prenatal and neonatal exposure to EDCs, which leach from MPs products, may cause irreversible changes in the reproductive axis and central nervous system of the offspring of various species [11]. Moreover, MPs exposure during the neonatal period is linked to the development of multiple illnesses in adulthood [11]. Through a Raman microspectroscopy analysis, a recent study demonstrated that MP fragments can be distributed in a human placenta sample [12]. More surprisingly, through the placenta, the fragments are also distributed and accumulated in the fetus, which may have the potential to cause harmful effects later in life. Therefore, the present study aims to summarize current evidence regarding the effects of MPs exposure on vital body systems, including the digestive, reproductive, central nervous, immune, and circulatory systems, during the early developmental stage. In addition, this study provides essential information regarding the possible emergence of various diseases later in life (i.e., adulthood).

## 2. Sources and Modes of MP Transmission

The primary sources of MPs are small plastic pellets or microbeads designed for commercial use, including cosmetic products and microfibers from clothing and other textiles. Meanwhile, the secondary source of MPs is defined as the breakdown of larger plastic particles, such as plastic bottles, into minute plastic particles (5 mm) through an exposure to environmental elements, such as sunlight and ocean waves. MPs are tiny, thus wastewater treatment technologies cannot filter them out; hence, they may enter and spread throughout rivers, seas, and freshwater supply systems [13]. Moreover, several possible pathways are available for transmitting MPs to a child, such as via placental transfer, the oral route, inhalation, breastfeeding, and dermal exposure [14].

**Figure 1:**
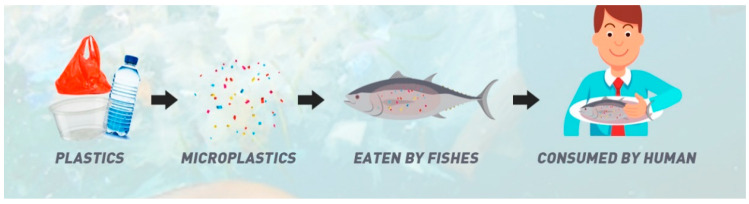
Sources and Modes of MPs Transmission. Illustration with permission from Jay Weaver © 2020 Design conscious [18].

Most living things accidentally consume MPs with their food sources in an ecosystem. Similarly, MPs may also enter the human body through the food chain. In a food chain, MPs are bioaccumulated from organisms at a lower trophic level to those at a higher trophic level through the food chain [15]. Therefore, a serious concern occurs when humans are at the highest trophic level, making them the highest bioaccumulator in the food chain [16]. Moreover, humans are also exposed to MPs from food and beverages contaminated with MPs during manufacturing [17] or to MPs leaking from numerous plastic packages. Figure 1 illustrates the flow of MPs materials that are bioaccumulated and transferred in a food chain that ends in humans.

MPs are also likely to be found in numerous food items due to their bioavailability and ubiquity in aquatic and terrestrial environments. For example, a previous study found that 81% of tap water from 159 countries has MPs particles [15]. Furthermore, scientific tests on polystyrene (PS) drinking cup lids showed that MPs and nanoplastics formed over time as the material broke down [15]. Various products also employ commercially generated MPs, which will ultimately become plastic garbage in the oceans and on land, making their way further into the food supply chain [15].

Recent research has revealed that the most significant mode of MPs transmission into the human body is through digestion or oral intake. Scientific evidence from a previous study found that MPs are being swallowed through food and drink intake [19]. In terms of exposure during the early developmental stage, milk from breastfeeding can transfer chemical metabolites from the mother to the offspring, because a child’s body load mirrors its mother’s exposure and correlates with the duration of nursing. Among toddlers, plastic toys and fabrics can also contribute to MPs exposure and related pollutants through tasting, licking, and chewing. Toys are mostly made up of plastic and also contain harmful plastic additives (such as EDCs and BPA) to obtain or optimize specific product properties [20]. Thus, toys may contribute as MPs sources where children commonly put the toys in their mouths due to their unique hand-to-mouth behavior. Zhang et al. [21] reported that infant feces had more significant quantities of polyethylene terephthalate (PET) MPs than adult feces. Unexpectedly, Li et al. [22] revealed that plastic infant bottles produced up to 16 million MPs per liter when shaken with warm water, and the sterilization process at high temperatures may also leach MPs from the bottles. Thus, studies on the safety of plastic usage during pregnancy and early infancy should be intensively conducted in the future to ensure minimum health risks during a child’s development.

Through the respiratory system, MPs can deeply penetrate the lungs, remain on the alveolar surface, and translocate to other body areas [15,23]. Depending on their size, plastic breakdown products may become airborne and inhalable [24]. Substantially more MPs, including synthetic fibers with polypropylene and PET polymers, prevail indoors. In the modern lifestyle era, many people tend to spend most of their time indoors [25]. Hence, assessing indoor inhalation exposure is crucial for understanding the potential health effects of MPs particles on the lung architecture and breathing rates and patterns of infants and children that change as they develop [14].

Varying amounts of MPs are found in numerous health and beauty products. For example, microbeads used in body and face scrubs are applied directly to the skin, which may introduce MPs into the body through dermal exposure. Other primary routes of MPs exposure are through medication administration or skin care routines via dermal absorption. Moreover, plastic particles may enter the body through sweat glands, skin wounds, or hair follicles. Compared with adult skin, children’s skin has the stratum corneum, the most superficial layer of skin that is thinner and less effective in preventing MPs intrusion [26]. Hair follicles, sweat glands [27], damaged skin, and atopic dermatitis (eczema) are points of entry for small particles less than 100 µm [28]. Child exposure to MPs may occur via plastic packaging, emollients, and infant personal care items such as lotion, oil, and other hygiene chemical products [14].

## 3. Potential Risks of MPs Exposure during the Early Developmental Stage

Over the last few decades, research has shown that pregnancy and infancy are vulnerable periods to environmental toxins; however, the effects of plastic particle exposure during early periods of sensitivity are nearly completely unknown [29]. We can expect children to have a specific and unique exposure to MPs because their behavior is also unique. Activities such as crawling and hand-to-mouth action that reflect their developing motor skills contribute to differences in their exposure to the environment compared with adults [14,30]. Children are also more likely to be exposed to pollution due to their natural desire to try and explore new things, and their activities can be challenging to control at times; in fact, they are still unable to distinguish between that which is beneficial and harmful [14].

**Figure 2:**
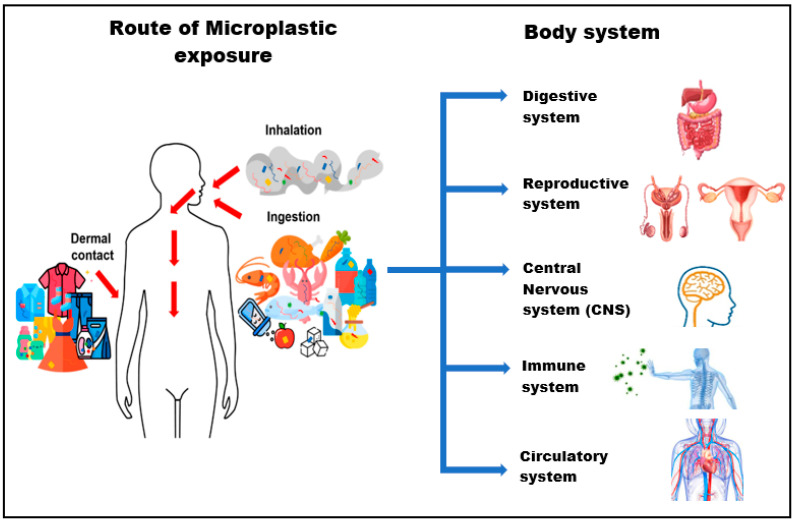
Human exposure to MPs via inhalation, ingestion, and dermal contact. MPs can be translocated to target systems, such as the digestive, reproductive, central nervous, immune, and circulatory systems, which may lead to toxicity effects. [34] © 2021 toxics.

Cox et al. [31] assessed MPs exposure on the human scale. On the basis of the consumption of food and drinking water in the United States, they estimated a daily MPs exposure of 203 particles for females and 223 particles for males. By comparison, Nor et al. [32] used a physiologically based pharmacokinetic model to simulate the lifetime accumulation of MPs and the projected daily consumption rates of 553 particles for children. However, in accordance with other pediatric studies, a single infant’s MPs consumption through feeding bottles ranges from 14,600 to 4,550,000 particles per day, with the lowest amounts found in Africa and Asia [22]. This vast range illustrates the significant uncertainty that surrounds human exposure to MPs, particularly during early life, and the considerable analytical hurdles associated with measuring MPs [33]. Thus, these greater exposure levels occur concurrently with the key development of the digestive, central nervous, reproductive, immune, circulatory, and other vital body systems. Figure 2 shows human exposure to MPs by inhalation, ingestion and dermal contact which may be translocated to vital body system.

### 3.1. Digestive System

Numerous animal investigations have revealed that ingested MPs accumulate in the guts of diverse species. MPs with a maximum size of more than 150 µm are not absorbed; instead, they are linked to the intestinal mucus layer and come into direct contact with the apical region of intestinal epithelial cells; meanwhile, smaller particles can pass through the mucus barrier, which may result in intestinal inflammation and local immune system consequences [35]. Notably, MPs in the air may affect the digestive tract and the immune system (absorbed by the pulmonary epithelium). Furthermore, MPs frequently enter the esophagus, stomach, and intestines through the mouth, producing a toxic effect on the digestive tract [36].

In another study, mice were exposed to various polyethylene (PE) MPs, causing inflammation and higher TLR4, AP-1, and IRF5 expression levels in the intestines of mice exposed to high amounts of MPs (Li et al., 2020) [6]. Furthermore, Deng et al. [37] found that a significant amount of MPs accumulated in the liver, kidneys, and gut of mice fed with pristine PS-MPs (5 µm and 20 µm) for 28 days, with larger particles dispersed regularly across all tissues and smaller particles found at a higher concentration inside the gut, implying that these particles will potentially impair the energy metabolism, lipid metabolism, oxidative stress level, and neurotoxic responses. However, a recent research found that MPs may reduce the secretion of intestinal mucus, increase the thiobarbituric acid (TBA) concentration in the liver, and cause metabolic disorders [38]. Moreover, the exposure of mice to PS-MPs for 28 days may decrease the lipid metabolism and cause a change in oxidative stress markers [39].

A previous study also found that pregnant mice exposed to MPs through ingestion developed gut microbiota dysbiosis, intestinal barrier dysfunction, and metabolic problems [21]. Immune cells convert molecular oxygen into reactive oxygen species during tissue hypoxia due to intestinal mucosa inflammation. Intestinal microbiota is a critical modulator of the mucosal redox potential. In addition, a continuous exposure to MPs particles causes dysbiosis of gut microbiota, intestinal barrier failure, and metabolic abnormalities in mice [22,38]. MPs have also been found in the feces of human volunteers, as reported by Schwabl et al. [40]. The wide variety of data gained from in vitro and animal studies have helped comprehend how ingested MPs breach the intestinal barrier, leading to the idea that probable harmful effects on the health of the human digestive system must be considered. Table 1 shows the summary of the effect of MPs exposure on the digestive system during the early developmental stage.

### 3.2. Reproductive System

Given their small size, EDCs in MPs may also interfere with human reproductive development by altering the normal secretion of the reproductive and gonadotropin hormones [41,42]. For example, BPA is one of the EDCs in plastic materials, and it is a toxic compound that may leach from numerous plastic products. Furthermore, studies have proven that fetuses, babies, and children are more sensitive to EDCs than adults due to the critical role of hormonal balance in growth and development [43]. In addition, many studies have demonstrated that EDCs cause permanent changes in the reproductive and central nervous system of the offspring of various species due to perinatal and neonatal exposure to these substances [11]. This finding implies that early life exposure to MPs or EDCs may increase a person’s susceptibility to illnesses [11].

Gonadal hormone inhibition at the level of the hypothalamic tonic center is more potent in childhood and diminishes with the advent of puberty. In reality, the extremely modest amount of hormones generated by the gonads can inhibit the release of the gonadotropin-releasing hormone (GnRH) and the production of gonadotropins during childhood. As puberty approaches, the sensitivity of the tonic center receptor to the activities of sex hormones decreases gradually [43]. Recent research has revealed that plastic particles in the placenta that comprises the chorioamnionitis membranes, the fetal side, and the maternal side [12,42], demonstrate that some interactions with the reproductive system may influence offspring viability [43]. MPs can affect multiple cellular regulatory pathways in the placenta, potentially leading to poor pregnancy outcomes, such as preeclampsia and fetal growth limitation [44]. In addition, a host’s defense mechanism regards MPs as foreign substances, which may result in local immunoreactions. A disturbance in immune balance can result in various early pregnancy issues, including spontaneous abortion, preeclampsia, fetal growth restriction, premature delivery, and stillbirth [45,46].

MPs exposure has also been reported to interfere with spermatogenesis and the GnRH levels of male rats, and cause metabolic diseases and dysplasia in the succeeding generation of mice [47]. Considerable evidence exists that EDC exposure at a critical stage of development may have long-term reproductive and carcinogenic effects. A group of scientists found that an in utero exposure to MPs at low and high doses in dam rodent animal models might induce the development of ovarian cysts and ovarian cyst-adenomas in their mature offspring (i.e., 18 months of age) [48]. Early exposure to bis (2-ethylhexyl) phthalate (DEHP) has also been found to have consequences on male pubertal development, including a shortened anogenital distance (AGD), areola and nipple retention, reduction in reproductive organ weight, undescended testes, and irreversibly incomplete preputial separation (separation of the prepuce from the glans penis).

MPs can also obstruct gamete binding by interfering with their plasma membrane fluidity; they may cover the embryo’s surface, causing hypoxia, or accumulate in the yolk sac, affecting nutrient absorption and causing abnormal offspring growth and development and metabolic disturbances [36]. In accordance with Barakat et al. [49], prenatal exposure to DEHP (GD11-birth) reduced fertility in male mice and lowered testosterone, serum estrogen, and luteinizing hormone (LH) levels. Animals that are prenatally exposed to DEHP may also have various gonadal and epididymal abnormalities [49]. Furthermore, microparticle deposition due to a prolonged exposure to low levels of MPs during fetal development may influence offspring’s health for the rest of their lives [42]. Ma et al. [50] revealed that dosages of dibutyl phthalate (DBP) induced male developmental and reproductive damage in rats, including a decrease in AGD, histological damage to the testis, and seminiferous tubule cell death. Hence, the current study found that exposure to MPs exerts adverse effects on the ovary and may be a risk factor for female infertility, providing new insights into the toxicity of MPs on the female reproductive system [51]. Table 2 shows the summary of the effects of MPs exposure on the reproductive system during the early developmental stage.

### 3.3. Central Nervous System

MPs serve as a vector for the wide variety of EDCs, which may interfere with hormonal systems, particularly during the early life development of the prepubertal period. One study found that perinatal exposure to low levels of EDCs may cause developmental defects and long-term neurological consequences in offspring [52]. In addition, these EDCs cause cellular and molecular alterations in the central nervous system, which can result in behavioral, memory, learning, and neurodegenerative problems later in life [53].

Epidemiological studies have shown that exposure to EDCs during pregnancy and breastfeeding may increase the risk of anxiety, sadness, aggressiveness, depression, or attention-deficit/hyperactivity disorders throughout childhood or later in the pubertal phase [54]. All these risks and potential disruptions may be due to increased oxidative stress levels and mitochondrial instability within important brain areas. In addition, cognitive memory can also be affected by EDCs through the decreased expression of the *N*-methyl-d-aspartate receptor and the estrogen receptor beta [55], and the increased DNA methylation of the estrogen receptor gene inside the hippocampus throughout postnatal development [11,56].

In particular, BPA exposure throughout the early stages of life has been linked to several neurodevelopment abnormalities in children [11]. Lee et al. [57] investigated the effects of diastolic blood pressure exposure during pregnancy and lactation on offspring neurodevelopment. Their results revealed delayed neurodevelopment, an increase in the frequency of dark neurons, and dopamine receptor D2 gene expression in the cerebral cortex. These findings show that, regardless of the methodological technique used, exposure to phthalates throughout the early stages of life causes neurodevelopmental and behavioral consequences in maturity that may be passed on to future generations.

Neurotoxicity has also been reported in vivo following a persistent exposure to fine particles, particularly MPs, probably due to immune cell activation in the brain and oxidative stress. However, reducing excitatory to inhibitory synaptic density in the cerebral cortex and hippocampus of male mice aged 8 weeks resulted in neuronal damage [58]. Furthermore, PS-MPs exposure (5 µm and 20 µm) dramatically decreased acetylcholinesterase (AChE) activity in mice liver, indicating a high risk of neurotoxicity in mammals [37]. In addition, previous studies revealed that BPA may increase oxidative stress, resulting in mitochondrial dysfunction that affects the behavior and functioning of children with autism spectrum disorder (ASD) [59]. Moreover, the exposure of mice to diastolic blood pressure caused an alteration in the hippocampus, poor performance, and low memory retention [60]. Table 3 shows the summary of the effect of MPs exposure on the central nervous system during the early developmental stage.

### 3.4. Immune System

MPs may have an effect on the immune system due to their physicochemical features [47]. Park et al. [47] hypothesized that the cascade signaling of the stomach walls’ immune response was triggered by the physical stress of PE-MPs. Other first-line immune responses were also observed, such as IgA and neutrophils working together to protect the host from a repeated PE-MPs exposure. In addition, several pieces of evidence have shown that the bioaccumulation of MPs can impair metabolic equilibrium, and consequently, disrupt the immune system’s efficiency [38]. Furthermore, Hu et al. [42] revealed that PS-MPs exposure during gestation can harm pregnancy outcomes through immunological disruption. By contrast, the composition of lymphocytes in the spleen of pups from PE-MPs-treated dams was significantly altered; this condition may disrupt immunological balance, implying that toxicity effects are due to maternal toxicity exposure during pregnancy [47].

The placenta serves as the biological connection between the fetus and the environment. Thus, the existence of MPs in placental tissue necessitates a reimagining of the immune system’s self-tolerance pathway [12]. Once MPs are introduced into the human body, they may aggregate and exert a localized toxicity by activating or increasing immunological responses, lowering the defense mechanisms against infections, and affecting energy storage use [61]. Furthermore, MPs can affect various processes, including placental cellular regulatory processes, such as immunity mechanisms throughout pregnancy; growth factor signaling during and after implantation; unusual chemokine receptor activities that control the mother–fetal connection; and natural killer cells, T cells, and other immune cells throughout normal pregnancy. These disruptions may cause complications, including preeclampsia and fetal growth restrictions [44]. Moreover, Saravia et al. found in vivo that combustion-derived MPs may cause transient immunodeficiency in mice due to the increased production of anti-inflammatory cytokines, the inhibition of T helper cells, and the reduced generation of T effector cells [1,62].

Numerous changes in earlier research illustrate that plastics affect the immune system and underscore the necessity for further immunotoxicity research on animals that is more closely associated with humans. In mice, for example, 5 weeks of exposure to PE-MPs may alter the serum levels of interleukin-1 (IL-1) and the granulocyte colony-stimulating factor, lower the regulatory T cell population, and raise the fraction of Th17 cells in splenocytes [22]. In addition, MPs and DEHP also alter the composition of gut microbiota, with the relative abundance of some energy metabolism and immune function-related bacteria dramatically changing (Deng et al., 2020) [37]. Moreover, an exposure to plastic additives (e.g., BPA) impairs the immune response in adults by altering the synthesis of cytokines and antibodies by these cells, making them more susceptible to infections, particularly toxocariasis [63]. Table 4 shows the summary of the effects of MPs exposure on the immune system during the early developmental stage.

### 3.5. Circulatory System

The circulatory system consists of the heart, blood vessels, lymph, and glands that circulate blood and lymph throughout the body. Once MPs particles are swallowed into the digestive system, they will undergo various absorption and translocation mechanisms that are influenced by particle size, particle content, and the biology of the organism’s gastrointestinal (GI) tract; organisms range from a filter feeder to mammalian physiology [64]. After exposure, micro-sized particles are accumulated in the digestive tubules and translocated into the circulatory system. These small plastic particles are transported from the GI lumen to the follicles and then to the circulatory system through specialized microfold cells in Peyer’s patches via phagocytosis [65]. However, absorption was also detected in the large intestine, notably in areas with abundant lymphoid tissues (Lett et al., 2021) [64]. Hwang et al. [66] estimated that 1–4% of MPs that reached the colon would be translocated into the circulatory system. Another study found that around 10% of the PS particle dosage given to the rats was retrieved from their GI system [64].

The gut–vascular barrier may be compromised through these mechanisms, allowing MPs to enter the circulatory system and gain access to the liver through the portal vein [67]. Moreover, the accumulation of MPs in lung tissues can cause chronic pulmonary diseases [68]. Supposedly, a substantial number of such aggregated protein–plastic complexes make their way into the circulatory system. In such a case, they may cause blood artery obstruction and loading of red blood cells (RBCs) with plastic particles at a high ratio of 10:50, revealing RBC damage caused by mechanical, osmotic, and oxidative stresses [67]. Furthermore, DEHP elevates blood pressure in humans by raising angiotensin II levels [69]; thus, deducing that an increase in blood pressure causes mechanical stress on the glomerular walls, which can lead to damage, is plausible [70]. In addition, medical research on rats and humans has revealed that polyvinyl chloride [8] and PS [71] MPs smaller than 150 µm may quickly diffuse from the GI cavity to the lymph and circulatory systems [72].

Previous research found that MPs can accumulate in the tissues of 5-week-old mice and cause alterations of blood biomarkers [37]. Furthermore, Park et al. discovered that MPs may reduce neutrophils and IgA levels in the bloodstream [47]. Rongli et al. [73] recently revealed that PS-MPs exposure may cause hematotoxicity to a certain extent, decrease blood cell count, and induce oxidative stress in 5-week-old mice. In addition, PS-MPs have led to cardiovascular toxicity by inducing cardiac fibrosis, caused apoptosis of the myocardium via oxidative stress, and led to collagen proliferation in the heart that may result in circulatory system problems [22]. Hence, plastic particles, such as PE, can also contribute to RBC aging, their removal from circulation, and impair RBCs’ oxygen transport [74]. Meanwhile, one study found that MPs may cause cardiovascular disease by altering gene transcription and upregulating the ventricle and atrium of the heart by using a monkey as a sample [75]. Table 5 shows the summary of the effects of MPs exposure on the circulatory system during the early developmental stage.

## 4. Limitations

Several studies have been conducted on the MPs exposure of aquatic and marine organisms. However, research on the risk assessment of MPs during early development is minimal, particularly on mammals and humans. The assessment of human risk exposure to MPs remains a research gap due to the lack of validated methodologies, approved reference materials, and uniformity across the employed analytical processes. Given the wide range of particle size, shape, and chemical composition of plastics, the potentially dangerous consequences of various forms of MPs on mammal and human health remain unclear. In addition, the animal models used in previous research to reveal the effects of MPs on humans are highly limited. More studies should be conducted using other species of mammal models, such as rabbit, birds, swine, and monkey.

## 5. Future Recommendations

Considering all the potential effects of MP exposure on humans, particularly during their critical development stage, conducting extensive studies on the possible effects of MPs exposure on human and mammal animal models is urgently necessary. Extensive scientific findings will be used to bring awareness to all stakeholders, including the legislative body, the public, the education sector, and industries. Furthermore, enacting solid legislative laws and policies to manage the excessive use of plastic products is crucial; otherwise, the health of ecosystems and living organisms will inevitably deteriorate in the coming years. Consequently, future attention must be given to this specific issue to understand the cytotoxicity mechanism of MPs and defend its safety.

We feel that the government and industries must exert the most significant effort to protect children from MPs exposure. These procedures include avoiding plastic contact of children’s meals, regular wet cleaning of the house, and the careful selection of safe personal care items and building materials. However, we advocate using the golden rule to guide policymakers’ approach to MPs and child health, given the uncertainties surrounding the risk of MPs exposure and its consequences throughout pregnancy and infancy. Simultaneously, governments must encourage research that will assist in understanding and quantifying the hazards of MPs. In this regard and on the basis of the most recent studies, we advocate for an increased surveillance of MPs in children’s settings.

Furthermore, we encourage the scientific community to collaborate across disciplines and outside academia to improve the understanding of early life MPs and plastic chemical exposure. We believe that such action is critical, because research on other pollutants has shown that we are living in sensitive times associated with adverse health effects from childhood to later life. Plastic pollution is a chronic and insidious problem that the environment is currently facing and will continue to face in the future. With no long-term remedy on the horizon, the risks, particularly to human health, are worth investigating and defining in greater depth.

## 6. Conclusions

In conclusion, although foundational research on children’s exposure to MPs is severely lacking, exposure to MPs and other plastic additives during the critical stages of life clearly induces numerous changes in the digestive, reproductive, central nervous, immune, and circulatory systems of a child. These changes may have different health effects on adults. Our review of the fragmented (but expanding) database around early life MPs exposure presents grounds for concern. Finally, existing research on the potential short- and long-term effects of MPs exposure on the health of children, teenagers, and adults should encourage researchers to delve deeper into the connections between environment and health. Research should focus on environmental health implications, and we must address these issues at the international, national, and local levels as soon as possible.

## Figures and Tables

**Table 1:** Summary of the effects of MPs exposure on the digestive system during the early developmental stage.

Organisms	Types of MPs	Size (µm) and Concentration	Age and Duration of Exposure	Consequences to Early Life Stages	References
ICR mice	PS-MPs	Size: 5 µm and 20 µm	Age: 5 weeks Exposure: 28 days	-MPs accumulate in kidneys, liver, and gut -Induce disturbance of energy -Induce disturbance of lipid metabolism -Oxidative stress	[37]
Mice	Fluorescent PS-MPs	Size: 5 µm and 20 µm Concentration: 0.2 mg/mL	Age: 5 weeks Exposure: 28 days	-Decrease lipid metabolism-associated biomarkers of TG and total cholesterol (TCH) level -Change in oxidative stress markers -Change in energy and lipid metabolism	[39]
ICR Mice	PS-MPs	Size: 0.5 µm and 5 µm Concentration: 100 µg/L and 1000 µg/L	Age: offspring 1 day Exposure: from gestational day 1 to birthday 1	-Decrease amino acid in female mouse offspring but increase it in male mouse offspring -Change in acyl-carnitine and free carnitine -Fatty acid metabolism disorder -Induce metabolic disorders in offspring	[21]
Mice (C57BL/6)	PE-MPs	Size: — Concentration: 6, 60, and 600 µg/day	Age: early exposure Exposure: 5 weeks	-Inflammation development -Decrease the percentage of Th17 and Trey -Inflammation to the intestine (duodenum and colon) -Induce intestinal dysbacteriosis	[22]
ICR mice	Pristine and fluorescent PS-MPs	Size: <5 µm Concentration: —	Age: 5 weeks Exposure: 6 weeks	-Reduce intestinal mucus secretion -Cause damage to the intestinal barrier function -Decrease actinobacteria content -Cause metabolic disorder -Alter the structure of gut microbiota in cecal contents -Impair intestinal barrier function -Increase TBA in the liver -Effect on feeding behavior and growth rate	[38]

**Table 2:** Summary of the effects of MPs exposure on the reproductive system during the early developmental stage.

Organisms	Types of MPs	Size (µm) and Concentration	Age and Duration of Exposure	Summary of Findings	References
ICR mice	PE-MPs	Size: — Concentration: 0.125, 0.5, and 2 mg/day/mouse	Age: 6 days (male and female) Exposure: 90 days	-Alter the number of live births -Decrease the sex ratio of pups -Induce damage to various tissues -Induce clinical and pathological changes -Alter growth and reproduction and increase the number of abnormal neonates	[47]
Mice	BPA (plastic additives)	Size: — Concentration: 0.1, 1, 10, 100, and 1000 µg/kg/day	Age: mice offspring 2 days after delivery Exposure: from Day 0 (pregnancy) to delivery	-Ovarian cyst -Structural (prenatal exposure) and cellular (neonatal exposure) alterations -Altered HOX gene expression during the differentiation of the reproductive tract -Oviductal alterations -Weak Erα binding -Uterus: Increase in stromal polyps -Transform SHE cells and induce aneuploidy	[48]
Male mice	DEHP	Size: 20, 200, and 500 µm Concentration: 750 mg/kg/day	Age: day 1 after birth Exposure: gestation day 11 until birth	-Gonadal dysfunction in offspring -Reduce fertility -Low serum testosterone, high estradiol, and high LH level -Epididymal abnormalities -Induce premature reproductive senescence	[49]
Rats	DBP (plastic additives)	Size: — Concentration: 50, 250, and 500 mg/kg/day	Age: 21 days Exposure: gestation day 14 until day 18 after birth	-Male development and reproduction toxicity -Decrease in AGD -Histological damage of the testis -Apoptosis of seminiferous tubule cells -Disruption of the expression of Rasd1 and MEKY2 and the Bcl-2/Bax ratio	[50]
Mice (57BL/6)	Saline of PS-MPs	Size: 5.0–5.9 µm Concentration: 0.1 mg/day	Age: 5 weeks Exposure: 30–40 days	-Change in the sex ratio of offspring -NLRP3/Caspase-1 signaling pathway -Decrease ovarian reserve -Decrease the number of total follicles and ovary size	[51]

**Table 3:** Summary of the effects of MPs exposure on the central nervous system during the early developmental stage.

Organisms	Types of MPs	Size (µm) and Concentration	Age and Duration of Exposure	Consequences to Early Life Stages	References
Mice	DBP	Size: — Concentration: 0, 50, and 100 mg/kg/day	Age: offspring Exposure: gestation day 13 to postnatal day 15	-Reduction in the protein expression levels of Nr4a3, Egr1, Arc, and BDNF, and the phosphorylation of AKT -Decrease scores in negative geotaxis at PND 7 and swimming scores and olfactory orientation tests at PND 14 -Increase dark neurons -Delay pup development	[57]
Inbred Swiss albino mice	BPA (plastic additives)	Size: — Concentration: 50 µg/kg/day	Age: 21 days Exposure: 3 weeks and 8 weeks	-Anxiety-like behavior -Alterations in the ratio of excitatory–inhibitory proteins -Inhibited PSD95 expression in the cerebral cortex and hippocampus -Reduce morphological changes, spine stability, and blocked LTP induction	[58]
Mice	Pristine PS-MPs	Size: 5 µm and 20 µm Concentration: -	Age: 5 weeks Exposure: 28 days	-Increase activity of AChE: reduction in cholinergic neurotransmission efficiency -Increase threonine, aspartate, and taurine in serum (neurotransmitter substance) -Reduce phenylalanine	[37]
Human (infants)	BPA (plastic additives)	No data	Age: 6 years Exposure: no data	-Increase oxidative stress -Cause mitochondrial dysfunction that will disturb the function and behavior of children with ASD	[59]
Winstar stain rats	DBP (plastic additives)	Size: — Concentration: 500 mg/kg BW	Age: no data Exposure: gestation days 6 to 21 (3 weeks lactation)	-Changes in sensory motor development reflex response -Low memory retention -Alteration of cytoarchitecture in the hippocampus -Disrupt neural and endocrine functions	[60]

**Table 4:** Summary of the effects of MPs exposure on the immune system during the early developmental stage.

Organisms	Types of MPs	Size (µm) and Concentration	Age and Duration of Exposure	Consequences to Early Life Stages	References
ICR mice	PS-MPs	Size: 40–48 µm Concentration: 10 mg/kg/day	Age: 6 weeks Exposure: 90 days	-Induce immune response -Physical stress on stomach walls -Disrupt metabolic homeostasis -Alter composition of lymphocytes -Increase IgA concentration -Accumulation of damaged organelles	[47]
Mice (BALB/C and C57BL/6)	PS-MPs	Size: 10 µm Concentration: —	Age: 8–10 weeks (old mice) Exposure: during peri-implantation period	-Decrease percentage of decidual natural killer cells -Cytokine secretion shifts toward an immunosuppressive state -Decrease NK cells in the decidua -Disturb pro-inflammatory cells (T8 cells and M1-subtype macrophage) -Disturb pro-inflammatory cytokinase (IL-2, IL-6, TNF-α, and IFN-γ) -Oxidative stress to immune cells	[42]
C57BL/6 mice	PE-MPs	Size: No data Concentration: 6, 60, and 600 µg/day	Age: early exposure Exposure: 5 weeks	-Decrease the percentage of Th17 and Treg cells among CD4^+^ cells -Induce inflammation and higher TLR4, AP-1, and IRF5 expression -Induce intestinal dysbacteriosis and inflammation	[22]
Mice *(Mus musculus)*	PE-MPs	Size: 45–53 µm Concentration: –	Age: 5 weeks Exposure: 30 days	-Dysfunction of the intestinal epithelial barrier -Alteration of gut microbiota, which leads to abnormal immune response -Change in genera of important microbes that are essential for energy metabolism and immune function (e.g., *Butyricimonas*, *Lactobacillus*, and *Ruminococcus*)	[37]
Wistar rats	BPA (plastic additives)	Size: 45–53 µm Concentration: 250 µg/kg/day	Age: postnatal Exposure: day 5 of pregnancy to day 21 postnatal	-Decrease in the production of specific antibodies -Downregulate Th2 cytokines (IL-4, IL-5, and IL-13), and upregulate Th1 cytokines (IFN-γ and TNF-α) -Affect the performance of immune response during adult life -Abnormal cytokine and antibody production	[63]

**Table 5:** Summary of the effects of MPs exposure on the circulatory system during the early developmental stage.

Organisms	Types of MPs	Size (µm) and Concentration	Age and Duration of Exposure	Consequences to Early Life Stages	References
C57BL/6 mice	PS-MPs	Size: 5 µm Concentration: 0.1 mg and 0.5 mg	Age: 5 weeks Exposure: 28 days	-Decrease white blood cell count -Increase pit count -Induce oxidative stress -Inhibit the colony-forming ability of bone marrow cells -Damage blood system	[73]
Male Wistar rats	PS-MPs	Size: 0.5 µm Concentration: 0.5, 5, and 50 mg/L	Age: offspring Exposure: 90 days	-Lead to collagen proliferation of the heart -Induce oxidative stress -Activate the cardiac fibrosis-related Wnt/β-catenin signaling pathway -Lead to cardiovascular toxicity -Damage structure	[22]
Mice	PE	Size: 200 nm Concentration: —	Age: offspring	-Increase lipid peroxidation and alter membrane structure -Contribute to RBC aging and removal from circulation -Impair RBC for oxygen transport -Ability of PE particles to circulate naturally for long times in the bloodstream -Transfer PE reversibly to the pulmonary vasculature via RBC carriage	[74]
Monkey	BPA	Size: — Concentration: 400 µg/kg bw		-Effect of cardiovascular fitness -Alter transcription of genes that are reorganized for their role in cardiac pathophysiology -Upregulate ventricles and the right atrium of the heart	[75]

## Data Availability

Not applicable.

## References

[1] Rahman A., Sarkar A., Yadav O.P., Achari G., Slobodnik J. (2021). Potential human health risks due to environmental exposure to nano- and microplastics and knowledge gaps: A scoping review. Sci. Total Environ..

[2] Ali I., Cheng Q., Ding T., Yiguang Q., Yuechao Z., Sun H., Peng C., Naz I., Li J., Liu J. (2021). Micro- and nanoplastics in the environment: Occurrence, detection, characterization and toxicity—A critical review. J. Clean. Prod..

[3] O’Connor J., Mahon A.M., Ramsperger A., Trotter B., Redondo-Hasselerharm P., Koelmans A., Lally H., Murphy S. (2019). Microplastics in Freshwater Biota: A Critical Review of Isolation, Characterization, and Assessment Methods. Glob. Chall..

[4] Zhu M., Chernick M., Rittschof D., Hinton D.E. (2020). Chronic dietary exposure to polystyrene microplastics in maturing Japanese medaka (Oryzias latipes). Aquat. Toxicol..

[5] Ferreira I., Venâncio C., Lopes I., Oliveira M. (2019). Nanoplastics and marine organisms: What has been studied?. Environ. Toxicol. Pharmacol..

[6] Li D., Shi Y., Yang L., Xiao L., Kehoe D.K., Gun’ko Y.K., Boland J.J., Wang J.J. (2020). Microplastic release from the degradation of polypropylene feeding bottles during infant formula preparation. Nat. Food.

[7] Visalli G., Facciolà A., Pruiti Ciarello M., De Marco G., Maisano M., Di Pietro A. (2021). Acute and Sub-Chronic Effects of Microplastics (3 and 10 µm) on the Human Intestinal Cells HT-29. Int. J. Environ. Res. Public Health.

[8] Street M.E., Bernasconi S. (2021). Microplastics, environment and child health. Ital. J. Pediatr..

[9] Lucaccioni L., Trevisani V., Marrozzini L., Bertoncelli N., Predieri B., Lugli L., Berardi A., Iughetti L. (2020). Endocrine-Disrupting Chemicals and Their Effects during Female Puberty: A Review of Current Evidence. Int. J. Mol. Sci..

[10] Carroquino M.J., Posada M., Landrigan P.J., Laws E.A. (2013). Environmental Toxicology: Children at Risk. Environmental Toxicology: Selected Entries from the Encyclopedia of Sustainability Science and Technology.

[11] Solleiro-Villavicencio H., Gomez-De León C.T., Del Río-Araiza V.H., Morales-Montor J. (2020). The detrimental effect of microplastics on critical periods of development in the neuroendocrine system. Birth Defects Res..

[12] Ragusa A., Svelato A., Santacroce C., Catalano P., Notarstefano V., Carnevali O., Papa F., Rongioletti M.C.A., Baiocco F., Draghi S. (2021). Plasticenta: First evidence of microplastics in human placenta. Environ. Int..

[13] Vance M.E., Kuiken T., Vejerano E.P., McGinnis S.P., Hochella M.F., Rejeski D., Hull M.S. (2015). Nanotechnology in the real world: Redeveloping the nanomaterial consumer products inventory. Beilstein J. Nanotechnol..

[14] Sripada K., Wierzbicka A., Abass K., Grimalt J.O., Erbe A., Röllin H.B., Weihe P., Díaz G.J., Singh R.R., Visnes T. (2022). A Children’s Health Perspective on Nano- and Microplastics. Environ. Health Perspect..

[15] Yee M.S., Hii L.W., Looi C.K., Lim W.M., Wong S.F., Kok Y.Y., Tan B.K., Wong C.Y., Leong C.O. (2021). Impact of Microplastics and Nanoplastics on Human Health. Nanomaterials.

[16] Santillo D., Miller K., Johnston P. (2017). Microplastics as contaminants in commercially important seafood species. Integr. Environ. Assess. Manag..

[17] Karami A., Golieskardi A., Keong Choo C., Larat V., Galloway T.S., Salamatinia B. (2017). The presence of microplastics in commercial salts from different countries. Sci. Rep..

[18] Weaver J. Microplastics Found in Human Placentas. https://designedconscious.com/plastics-in-the-ocean/sustainability-news-stories/microplastics-found-in-human-placentas/.

[19] Akhbarizadeh R., Moore F., Keshavarzi B. (2019). Investigating microplastics bioaccumulation and biomagnification in seafood from the Persian Gulf: A threat to human health?. Food Addit. Contam. Part A Chem. Anal. Control. Expo. Risk Assess..

[20] Aurisano N., Huang L., Milà i Canals L., Jolliet O., Fantke P. (2021). Chemicals of concern in plastic toys. Environ. Int..

[21] Luo T., Zhang Y., Wang C., Wang X., Zhou J., Shen M., Zhao Y., Fu Z., Jin Y. (2019). Maternal exposure to different sizes of polystyrene microplastics during gestation causes metabolic disorders in their offspring. Environ. Pollut..

[22] Li B., Ding Y., Cheng X., Sheng D., Xu Z., Rong Q., Wu Y., Zhao H., Ji X., Zhang Y. (2020). Polyethylene microplastics affect the distribution of gut microbiota and inflammation development in mice. Chemosphere.

[23] Hathaway Q.A., Nichols C.E., Shepherd D.L., Stapleton P.A., McLaughlin S.L., Stricker J.C., Rellick S.L., Pinti M.V., Abukabda A.B., McBride C.R. (2017). Maternal-engineered nanomaterial exposure disrupts progeny cardiac function and bioenergetics. Am. J. Physiol.-Heart Circ. Physiol..

[24] Amato-Lourenço L.F., Dos Santos Galvão L., de Weger L.A., Hiemstra P.S., Vijver M.G., Mauad T. (2020). An emerging class of air pollutants: Potential effects of microplastics to respiratory human health?. Sci. Total Environ..

[25] Brasche S., Bischof W. (2005). Daily time spent indoors in German homes—Baseline data for the assessment of indoor exposure of German occupants. Int. J. Hyg. Environ. Health.

[26] Lehner R., Weder C., Petri-Fink A., Rothen-Rutishauser B. (2019). Emergence of Nanoplastic in the Environment and Possible Impact on Human Health. Environ. Sci. Technol..

[27] Alvarez-Román R., Naik A., Kalia Y.N., Guy R.H., Fessi H. (2004). Skin penetration and distribution of polymeric nanoparticles. J. Control. Release.

[28] Biagini Myers J.M., Khurana Hershey G.K. (2010). Eczema in early life: Genetics, the skin barrier, and lessons learned from birth cohort studies. J. Pediatr..

[29] Senathirajah K., Attwood S., Bhagwat G., Carbery M., Wilson S., Palanisami T. (2021). Estimation of the mass of microplastics ingested—A pivotal first step towards human health risk assessment. J. Hazard. Mater..

[30] Moya J., Bearer C.F., Etzel R.A. (2004). Children’s behavior and physiology and how it affects exposure to environmental contaminants. Pediatrics.

[31] Cox K.D., Covernton G.A., Davies H.L., Dower J.F., Juanes F., Dudas S.E. (2019). Human Consumption of Microplastics. Environ. Sci. Technol..

[32] Mohamed Nor N.H., Kooi M., Diepens N.J., Koelmans A.A. (2021). Lifetime Accumulation of Microplastic in Children and Adults. Environ. Sci. Technol..

[33] Williams A.T., Rangel-Buitrago N. (2022). The past, present, and future of plastic pollution. Mar. Pollut. Bull..

[34] Pironti C., Ricciardi M., Motta O., Miele Y., Proto A., Montano L. (2021). Microplastics in the Environment: Intake through the Food Web, Human Exposure and Toxicological Effects. Toxics.

[35] Hirt N., Body-Malapel M. (2020). Immunotoxicity and intestinal effects of nano- and microplastics: A review of the literature. Part. Fibre Toxicol..

[36] Yin K., Wang Y., Zhao H., Wang D., Guo M., Mu M., Liu Y., Nie X., Li B., Li J. (2021). A comparative review of microplastics and nanoplastics: Toxicity hazards on digestive, reproductive and nervous system. Sci. Total Environ..

[37] Deng Y., Zhang Y., Lemos B., Ren H. (2017). Tissue accumulation of microplastics in mice and biomarker responses suggest widespread health risks of exposure. Sci. Rep..

[38] Jin Y., Lu L., Tu W., Luo T., Fu Z. (2019). Impacts of polystyrene microplastic on the gut barrier, microbiota and metabolism of mice. Sci. Total Environ..

[39] Yang Y.F., Chen C.Y., Lu T.H., Liao C.M. (2019). Toxicity-based toxicokinetic/toxicodynamic assessment for bioaccumulation of polystyrene microplastics in mice. J. Hazard. Mater..

[40] Schwabl P., Köppel S., Königshofer P., Bucsics T., Trauner M., Reiberger T., Liebmann B. (2019). Detection of Various Microplastics in Human Stool: A Prospective Case Series. Ann. Intern. Med..

[41] Liu Z., Zhuan Q., Zhang L., Meng L., Fu X., Hou Y. (2022). Polystyrene microplastics induced female reproductive toxicity in mice. J. Hazard. Mater..

[42] Hu J., Qin X., Zhang J., Zhu Y., Zeng W., Lin Y., Liu X. (2021). Polystyrene microplastics disturb maternal-fetal immune balance and cause reproductive toxicity in pregnant mice. Reprod. Toxicol..

[43] Leonardi A., Cofini M., Rigante D., Lucchetti L., Cipolla C., Penta L., Esposito S. (2017). The Effect of Bisphenol A on Puberty: A Critical Review of the Medical Literature. Int. J. Environ. Res. Public Health.

[44] Ilekis J.V., Tsilou E., Fisher S., Abrahams V.M., Soares M.J., Cross J.C., Zamudio S., Illsley N.P., Myatt L., Colvis C. (2016). Placental origins of adverse pregnancy outcomes: Potential molecular targets: An Executive Workshop Summary of the Eunice Kennedy Shriver National Institute of Child Health and Human Development. Am. J. Obstet. Gynecol..

[45] Aneman I., Pienaar D., Suvakov S., Simic T.P., Garovic V.D., McClements L. (2020). Mechanisms of Key Innate Immune Cells in Early- and Late-Onset Preeclampsia. Front. Immunol..

[46] Ticconi C., Pietropolli A., Di Simone N., Piccione E., Fazleabas A. (2019). Endometrial Immune Dysfunction in Recurrent Pregnancy Loss. Int. J. Mol. Sci..

[47] Park E.J., Han J.S., Park E.J., Seong E., Lee G.H., Kim D.W., Son H.Y., Han H.Y., Lee B.S. (2020). Repeated-oral dose toxicity of polyethylene microplastics and the possible implications on reproduction and development of the next generation. Toxicol. Lett..

[48] Newbold R.R., Jefferson W.N., Padilla-Banks E. (2009). Prenatal exposure to bisphenol a at environmentally relevant doses adversely affects the murine female reproductive tract later in life. Environ. Health Perspect..

[49] Barakat R., Lin P.P., Rattan S., Brehm E., Canisso I.F., Abosalum M.E., Flaws J.A., Hess R., Ko C. (2017). Prenatal Exposure to DEHP Induces Premature Reproductive Senescence in Male Mice. Toxicol. Sci..

[50] Ma T., Yin X., Han R., Ding J., Zhang H., Han X., Li D. (2017). Effects of In Utero Exposure to Di-n-Butyl Phthalate on Testicular Development in Rat. Int. J. Environ. Res. Public Health.

[51] Hou J., Lei Z., Cui L., Hou Y., Yang L., An R., Wang Q., Li S., Zhang H., Zhang L. (2021). Polystyrene microplastics lead to pyroptosis and apoptosis of ovarian granulosa cells via NLRP3/Caspase-1 signaling pathway in rats. Ecotoxicol. Environ. Saf..

[52] Rachoń D. (2015). Endocrine disrupting chemicals (EDCs) and female cancer: Informing the patients. Rev. Endocr. Metab. Disord..

[53] DeBartolo D., Jayatilaka S., Yan Siu N., Rose M., Ramos R.L., Betz A.J. (2016). Perinatal exposure to benzyl butyl phthalate induces alterations in neuronal development/maturation protein expression, estrogen responses, and fear conditioning in rodents. Behav. Pharmacol..

[54] Ejaredar M., Lee Y., Roberts D.J., Sauve R., Dewey D. (2017). Bisphenol A exposure and children’s behavior: A systematic review. J. Expo. Sci. Environ. Epidemiol..

[55] Xu X.H., Zhang J., Wang Y.M., Ye Y.P., Luo Q.Q. (2010). Perinatal exposure to bisphenol-A impairs learning-memory by concomitant down-regulation of N-methyl-D-aspartate receptors of hippocampus in male offspring mice. Horm. Behav..

[56] Chang H., Wang M., Xia W., Chen T., Huo W., Mao Z., Zhu Y., Li Y., Xu S. (2016). Perinatal exposure to low-dose bisphenol A disrupts learning/memory and DNA methylation of estrogen receptor alpha in the hippocampus. Toxicol. Res..

[57] Lee S.M., Jeon S., Jeong H.J., Kim B.N., Kim Y. (2020). Dibutyl phthalate exposure during gestation and lactation in C57BL/6 mice: Maternal behavior and neurodevelopment in pups. Environ. Res..

[58] Kumar D., Thakur M.K. (2017). Anxiety like behavior due to perinatal exposure to Bisphenol-A is associated with decrease in excitatory to inhibitory synaptic density of male mouse brain. Toxicology.

[59] Metwally F.M., Rashad H., Zeidan H.M., Kilany A., Abdol Raouf E.R. (2018). Study of the Effect of Bisphenol A on Oxidative Stress in Children with Autism Spectrum Disorders. Indian J. Clin. Biochem..

[60] Piler M.B., Radha M.J. (2020). Gestational and lactational exposition to di-n-butyl phthalate increases neurobehavioral perturbations in rats: A three generational comparative study. Toxicol. Rep..

[61] Wright S.L., Kelly F.J. (2017). Plastic and Human Health: A Micro Issue?. Environ. Sci. Technol..

[62] Saravia J., You D., Thevenot P., Lee G.I., Shrestha B., Lomnicki S., Cormier S.A. (2014). Early-life exposure to combustion-derived particulate matter causes pulmonary immunosuppression. Mucosal Immunol..

[63] Del Río-Araiza V.H., Palacios-Arreola M.I., Nava-Castro K.E., Pérez-Sánchez N.Y., Ruíz-Manzano R., Segovia-Mendoza M., Girón-Pérez M.I., Navidad-Murrieta M.S., Morales-Montor J. (2020). Perinatal exposure to bisphenol A increases in the adulthood of the offspring the susceptibility to the human parasite Toxocara canis. Environ. Res..

[64] Lett Z., Hall A., Skidmore S., Alves N.J. (2021). Environmental microplastic and nanoplastic: Exposure routes and effects on coagulation and the cardiovascular system. Environ. Pollut..

[65] Gopinath P.M., Saranya V., Vijayakumar S., Mythili Meera M., Ruprekha S., Kunal R., Pranay A., Thomas J., Mukherjee A., Chandrasekaran N. (2019). Assessment on interactive prospectives of nanoplastics with plasma proteins and the toxicological impacts of virgin, coronated and environmentally released-nanoplastics. Sci. Rep..

[66] Hwang J., Choi D., Han S., Jung S.Y., Choi J., Hong J. (2020). Potential toxicity of polystyrene microplastic particles. Sci. Rep..

[67] Yong C.Q.Y., Valiyaveettil S., Tang B.L. (2020). Toxicity of Microplastics and Nanoplastics in Mammalian Systems. Int. J. Environ. Res. Public Health.

[68] Mattsson K., Johnson E.V., Malmendal A., Linse S., Hansson L.-A., Cedervall T. (2017). Brain damage and behavioural disorders in fish induced by plastic nanoparticles delivered through the food chain. Sci. Rep..

[69] Segovia-Mendoza M., Nava-Castro K.E., Palacios-Arreola M.I., Garay-Canales C., Morales-Montor J. (2020). How microplastic components influence the immune system and impact on children health: Focus on cancer. Birth Defects Res..

[70] Ichimura M., Kato S., Tsuneyama K., Matsutake S., Kamogawa M., Hirao E., Miyata A., Mori S., Yamaguchi N., Suruga K. (2013). Phycocyanin prevents hypertension and low serum adiponectin level in a rat model of metabolic syndrome. Nutr. Res..

[71] Waring R.H., Harris R.M., Mitchell S.C. (2018). Plastic contamination of the food chain: A threat to human health?. Maturitas.

[72] Browne M.A., Dissanayake A., Galloway T.S., Lowe D.M., Thompson R.C. (2008). Ingested microscopic plastic translocates to the circulatory system of the mussel, *Mytilus edulis* (L). Environ. Sci. Technol..

[73] Sun R., Xu K., Yu L., Pu Y., Xiong F., He Y., Huang Q., Tang M., Chen M., Yin L. (2021). Preliminary study on impacts of polystyrene microplastics on the hematological system and gene expression in bone marrow cells of mice. Ecotoxicol. Environ. Saf..

[74] Pan D., Vargas-Morales O., Zern B., Anselmo A.C., Gupta V., Zakrewsky M., Mitragotri S., Muzykantov V. (2016). The Effect of Polymeric Nanoparticles on Biocompatibility of Carrier Red Blood Cells. PLoS ONE.

[75] Chapalamadugu K.C., Vandevoort C.A., Settles M.L., Robison B.D., Murdoch G.K. (2014). Maternal bisphenol a exposure impacts the fetal heart transcriptome. PLoS ONE.

